# How to Evaluate Investment Efficiency of Environmental Pollution Control: Evidence from China

**DOI:** 10.3390/ijerph19127252

**Published:** 2022-06-13

**Authors:** Xiaochun Zhao, Laichun Long, Qun Sun, Wei Zhang

**Affiliations:** 1 School of Management, Anhui University, Hefei 230601, China; 14006@ahu.edu.cn (X.Z.); l21201027@stu.ahu.edu.cn (L.L.); sunqun320@126.com (Q.S.); 2 School of Public Administration, Sichuan University, Chengdu 610065, China

**Keywords:** environmental pollution control investment efficiency, three-stage DEA, super-efficient SBM, Global-Malmquist-Luenberger index, regional differences

## Abstract

Clarifying the efficiency of investment in environmental pollution control is conducive to better control of environmental pollution. Based on panel data of 30 provinces and cities in China from 2008 to 2017, this study combines the three-stage super-efficient SBM-DEA model and the Global-Malmquist-Luenberger index to measure the efficiency of investment in environmental pollution control in China and analyze regional differences. The results show that: First, the investment efficiency of environmental pollution control in China shows a rising trend year by year, but there are significant differences among provinces and regions; the presence of random factors and environmental variables makes the control efficiency underestimated. Second, excluding the effects of both, the national investment efficiency of environmental pollution control has improved significantly, but still has not reached the optimal effect; the gap between provinces and regions has narrowed while the investment efficiency of environmental pollution control has improved, and there is still an unbalanced situation. Third, the main driver of the year-on-year improvement in China’s environmental pollution control efficiency is technological progress; compared with northeastern China, technological progress has a more significant role in promoting eastern, central, and western China. Finally, based on the results, this paper focuses on making suggestions to promote environmental pollution control in China in terms of making regional cooperation, making good environmental protection investment and strengthening environmental protection technology research and development.

## 1. Introduction

Since the reform and opening up in 1978, China has experienced rapid economic growth. From a poor and backward developing country to become the second largest economy in the world and has the largest foreign exchange reserves in the world. However, the process of industrialization and urbanization has also brought serious environmental pollution problems [1], which is becoming increasingly serious and directly affects the daily lives of residents as well as the long-term development. Nearly half of China’s water sources cannot be used for drinking water, and the water quality of large reservoirs and lakes is generally poor [2]. The annual generation of industrial solid waste is among the highest in the world, and white pollution caused by plastic packaging and agricultural films can be seen everywhere [3,4]. While the economy is developing rapidly, it is urgent to seek a harmonious relationship between humans and the environment. It is noteworthy that the Chinese government has long been aware of the destruction of the environment due to various types of pollution, and a large amount of funds and various policies have gradually tilted toward the field of environmental pollution management. From 1981 to 2017, the investment in environmental protection increased from 2.5 billion yuan to 953.89 billion yuan. The increasing scale of environmental protection investment amount has failed to address the root cause of environmental pollution problems, and the effect of environmental pollution management in China is still not optimistic, with different degrees of environmental pollution problems in various provinces. However, the actual situation of the efficiency of investment in environmental pollution management in China’s regions and provinces is currently unclear. There are differences in environmental protection investment efforts, environmental protection awareness, and environmental protection policies among regions, and how to accurately evaluate the efficiency of their investment in environmental pollution control while taking into account these external differences is of great relevance to the Chinese government in formulating environmental protection policies.

With the rapid development of China’s economy, environmental pollution has become increasingly serious. The Chinese government also recognized this early and attached great importance to the treatment of environmental pollution, and continuously increased the investment in environmental pollution treatment [5]. Is China’s environmental pollution control investment efficiency gradually improving? China is a vast country with differences in economic volume and resource endowment in different regions, and environmental policies are supported differently in each region [6]. Are there regional differences in the efficiency of investment in environmental pollution control in China? Economically developed provinces have more investment in environmental protection, more research and development in environmental protection technology, higher education level and higher awareness of environmental protection, while economically backward provinces have relatively less investment in environmental protection [7]. Are there regional differences in the efficiency of investment in environmental pollution control in China? This paper takes 30 provinces and cities in China from 2008–2017 as the research object (due to the problem of missing data in Tibet, Hong Kong, Macao and Taiwan, they are excluded from the research object), selects input and output indicators by referring to scholars’ studies, and uses the super-efficient SBM model and the Global-Malmquist-Luenberger index to analyze the current situation of investment efficiency in environmental pollution control in China. The main research objectives of this study include the following: First, to evaluate the efficiency of investment in environmental pollution control in China; second, to clarify the changing trend of investment efficiency of environmental pollution control in China; then, comparing the differences in environmental pollution control efficiency among different regions; finally, identifying the main factors that affect the efficiency of environmental pollution control. Based on the research findings, relevant suggestions are made for environmental pollution control in China.

The arrangement of this paper is as follows: It first sorts out the related research about the efficiency of environmental pollution control. Afterwards, it introduces the research methods and data sources of this paper. Next, it displays and discusses the specific research results. Finally, it puts forward the research findings and corresponding policy recommendations and summarizes the shortcomings of this paper.

## 2. Literature Review

The research about the efficiency of environmental pollution control can be traced back to the end of the last century. Scholars used different methods to study the efficiency of environmental pollution control from various perspectives. In terms of research methods, they can be divided into two main categories. The first category is Data Envelopment Analysis (DEA) based on inputs and outputs. Reinhard (2000) was one of the first scholars to apply environmental variables to DEA input-output analysis, and they used the DEA method to measure environmental efficiency on dairy farms in the Netherlands and explained that the DEA method is capable of calculating any form of environmental efficiency score [8]. Mandal, S.K et al. (2010) used DEA analysis to estimate the environmental efficiency of the Indian cement industry by using the combustion of coal for cement production to produce a large amount of carbon dioxide as an undesired output [9]. Cecchini et al. (2018) used the DEA model and combined the results of life-cycle analysis to estimate the environmental efficiency and CO_2_ reduction potential of 10 dairy farms in Umbria, Italy, proposing that the marginal emission reduction costs showed a positive correlation with the measured environmental efficiency scores [10]. Other scholars constructed a system of input-output indicators based on provincial panel data in China and used DEA to evaluate the efficiency of eco-environmental management in rural China [11]. The second category is Stochastic Frontier Analysis (SFA) for regression equation analysis. For example, some scholars estimated the input efficiency of industrial environmental management in China using SFA models based on data from three types of industrial wastes and explored the overall characteristics of input efficiency [12]. Other scholars used stochastic frontier analysis (SFA) models to analyze the effects of energy consumption and environmental pollution on the efficiency of technological innovation in industrial enterprises [13]. Moreover, some scholars have used the improved DEA method to measure the efficiency of environmental governance. Goto et al. (2014) used three modified data envelopment analysis (DEA) models to assess the operational and environmental efficiency of regional industries in Japan [14]. Xiao S et al. (2019) conducted a study on environmental efficiency using three-stage DEA [15]. There is also Zou et al. who proposed to measure the regional environmental efficiency in China using non-desired outputs through the SBM model [16]. Among the methods to evaluate the efficiency of environmental pollution control, besides the two mainstream methods of DEA and SFA, other scholars have used methods to assess the economic efficiency of investments. For example, Akoto et al. (2020) used the net present value (NPV) and financial benefit-cost ratio (FBCR) methods to evaluate the environmental impact and economic profitability of several bioenergy sources [17]. Zhao et al. (2021) used the internal rate of return index (IRR) to study the waste treatment system in Beijing and made effective recommendations to improve the energy recovery efficiency of the waste treatment system [18].

The research results of these studies are beneficial to environmental pollution management, but they still have shortcomings. From the literature, it can be seen that the traditional DEA method and SFA method are the main choices of the pollution control efficiency evaluation method in the existing studies. First, these research methods adopted by scholars are either subject to large random errors and environmental factors, or they can only be scaled down equivalently in terms of input-output adjustment, which cannot guarantee the objectivity of efficiency evaluation results, or they only focus on analyzing investment efficiency of environmental pollution control at the static level. Second, there is a lack of research on the efficiency of environmental pollution control investment in different regions of China by combining the three-stage super-efficiency SBM-DEA model with the Malmquist index method. Therefore, in order to fill the above research gaps, this paper tries to combine these two approaches and conduct empirical analysis based on panel data of 30 provinces in China from 2008 to 2017, analyze the level of pollution control investment efficiency and its change characteristics in China from the overall level, and compare regional differences. Eastern China has a large population and the highest level of economic development. Central China is not as economically developed as Eastern China and has the second largest population, but has great development potential. Western China has a vast territory, the smallest population density, and the third highest level of economic development. In contrast, northeastern China has the smallest population and a smaller economy, but has a developed heavy industrial sector. In 2018, eastern China accounted for 51.75% of the national share of GDP and had 11 large cities (with a resident population of more than 5 million people); northeastern China, with 5.03% of the national GDP, had 4 large cities; central China, with 25.76% of the national GDP, had 3 large cities; and western China, with 17.10% of the national GDP, had 4 large cities [19,20]. Comparing the efficiency difference of environmental pollution control investment among different regions can provide policy recommendations for environmental pollution control in China.

## 3. Materials and Methods

### 3.1. Research Methods

#### 3.1.1. First Stage Super-Efficient SBM Model

The global super-efficient SBM (Slacks-based model) model with variable scale payoffs was proposed by Tone (2001) [21]. This model avoids the absence of feasible solutions and “technical regression” in the case of variable payoffs of scale; it ensures accurate efficiency results without the need for uniformity of scales. The equation is shown in (1).

(1) $$\rho =\min\frac{\frac{1}{m}\sum_{i=1}^{1}\frac{\bar{x}}{x_{ik}}}{\frac{1}{s_{1}+s_{2}}\left(\sum_{t=1}^{s_{1}}\frac{{\bar{y}}_{l}^{d}}{{\bar{y}}_{l_{0}}^{d}}+\sum_{k=1}^{s_{2}}\frac{{\bar{y}}_{k}^{u}}{{\bar{y}}_{k_{0}}^{u}}\right)}$$

$$s.t.\left\{ \begin{array}{l} \bar{x}\geq \sum_{j\neq 1,j\neq j_{0}}^{n}x_{ij}\lambda_{j};{\bar{y}}^{d}\leq \sum_{j=1,j\neq j_{0}}^{n}y_{l_{j}}^{d}\lambda ;{\bar{y}}^{u}\geq \sum_{j=1,j\neq j_{0}}^{n}y_{k_{j}}^{d}\lambda_{j}\\ {\bar{y}}^{d}\leq y_{l_{j}}^{d};{\bar{y}}^{u}\geq y_{k_{j}}^{d}\\ \lambda_{j}\geq 0,i=1,2,...,m;j=1,2,...,s_{1};k=1,2,...,s_{2} \end{array}\right.$$

where, *n* denotes the number of decision-making units (DMU), i.e., $y^{u}$ represents the number of provinces; *x* represents the data in the input index *m*; $y^{d}\;$ represents the data in the desired output index $s_{1}$; $y^{u\;}$ represents the data in the non-desired output index $s_{2}$; *λ* represents the weight of DMU; *ρ* denotes the efficiency value of environmental pollution control, *ρ* ≥ 1, which means the efficiency is relatively effective. Larger *ρ* represents the higher level of environmental pollution control.

#### 3.1.2. Second Stage Stochastic Frontier SFA Model

The stochastic frontier SFA (Stochastic Frontier Analysis) model proposed by Aigner, Lovell, and Schmidt (1977) [22] was used to quantify the errors and find the most influential stochastic factors and environmental errors. The true input and output values are calculated. With the input orientation, if there are *n* decision-making units (DMU), the initial slack value equation for *i* inputs of each DMU is shown in (2).

(2) $$S_{in}=f_{i}\left(Z_{n};\beta_{i}\right)+V_{in}+U_{in}$$

where *i* = 1, 2,..., *M*, *n* = 1, 2,..., *N*. represents the nth decision-making unit (DMU) observable external environmental variables; $\beta_{i}$ represents the parameter vector of external environmental factors; $f_{i}\left(Z_{n};\beta_{i}\right)$ represents the effect of external environmental variables on the input redundancy slack values, usually taken as $f_{i}\left(Z_{n};\beta_{i}\right)\;$*=* $\beta_{i}Z_{n}$; $V_{in\;}$ represents the effect of random factors, following $N\left(0,\sigma_{un}{}^{2}\right)$; $\;U_{in}$ represents the effect of internal management status and input size, following $N\left(0,\sigma_{un}{}^{2}\right)$; $V_{in\;}$ and $U_{in\;}$ are independent of each other. The regression results obtained from the calculation are used to adjust the input values of other relatively inefficient DMU, and Equation (3) is as follows:

(3) $${\left(X_{in}\right)}^{*}=X_{in}+\left[\max_{n}\left(\beta_{i}Z_{n}\right)-\beta_{i}Z_{n}\right]+\left[\max_{n}\left(V_{in}\right)-V_{in}\right]$$

where, $X_{in}$ represents the actual input value; ${\left(X_{in}\right)}^{*}$ denotes the adjusted input value; $\left[max_{n}\left(\beta_{i}Z_{n}\right)-\beta_{i}Z_{n}\right]$ represents that all decision-making units (DMU) are in a homogeneous environment; $\left[max_{n}\left(V_{in}\right)-V_{in}\right]\;$ represents the random error term that will be adjusted to the same condition for all DMU.

#### 3.1.3. The Third Stage after Adjustment Is again Substituted into the Super-Efficient SBM Model

It is brought into the global super-efficient scale payoff variable SBM model, i.e., Equation (1), for calculation to obtain more objective and accurate efficiency values and for empirical analysis.

#### 3.1.4. GML (Global-Malmquist-Luenberger) Index

The efficiency values measured by the super-efficient SBM are only static descriptions, while the GML index is a good complement to the SBM model and can dynamically analyze the changes in efficiency values between two years before and after. Therefore, the GML index proposed by Oh (2010) is introduced to measure the changes and influencing factors of governance efficiency in different periods and to conduct dynamic comparative analysis among regions [23]. The GML index can be further decomposed into the technical efficiency change index (EC index) and the technical progress change index (TC index), which respectively represent the contribution of technical efficiency improvement and technical improvement to the improvement of environmental pollution control efficiency in the period from *t* to *t* + 1 of the evaluation decision-making unit (DMU). The formula is shown in (4).

(4) $$\begin{array}{ll} GML(x^{t+1},y^{t+1},b^{t+1},x^{t},y^{t},b^{t}) & =\frac{E(x^{t+1},y^{t+1},b^{t+1})}{E(x^{t},y^{t},b^{t})}\\ & =\frac{E^{{}^{t+1}}(x^{t+1},y^{t+1},b^{t+1})}{E^{t}(x^{t},y^{t},b^{t})}\times \left[\frac{E(x^{t+1},y^{t+1},b^{t+1})}{E^{{}^{t+1}}(x^{t+1},y^{t+1},b^{t+1})}\times \frac{E^{t}(x^{t},y^{t},b^{t})}{E^{}(x^{t},y^{t},b^{t})}\right]\\ & =EC\times TC \end{array}$$

### 3.2. Variables Selection

At present, many studies have been carried out on the efficiency of environmental pollution management in academia, and on the basis of the excellent results of related scholars [24,25,26,27,28,29], considering the typicality, accessibility, and feasibility of relevant variable indicators, environmental pollution management is divided into three first-level indicators of input variables, output variables, and external environmental variables, and finally constructed into an indicator system containing 11 three-level indicators(See Table 1 for details).

### 3.3. Data Sources

This paper selects the data of 30 provinces and cities in China from 2008 to 2017 for the analysis (see Table 1 for detailed indicators). All data are from the official information released by the National Bureau of Statistics, mainly including the China Statistical Yearbook (2009–2018), China Environmental Statistical Yearbook (2009–2018) and the official information data of various provinces. Based on the exist studies and the situation of China’s economic and social development, the provinces covered in this study can be divided into four regions, the eastern region (Beijing, Tianjin, Hebei, Shanghai, Jiangsu, Zhejiang, Fujian, Shandong, Guangdong, Hainan), the northeastern region (Liaoning, Jilin, Heilongjiang,), the central region (Shanxi, Anhui, Jiangxi, Henan, Hubei, Hunan), and the western region (Inner Mongolia, Guangxi, Chongqing, Sichuan, Guizhou, Yunnan, Shaanxi, Gansu, Qinghai, Ningxia, Xinjiang) [30,31,32].

## 4. Results

### 4.1. First Stage Initial Super-Efficient SBM Analysis

**Figure 1:**
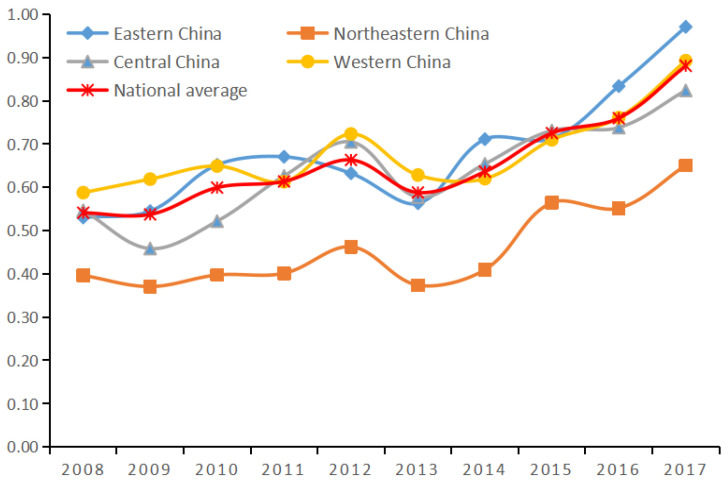
Change in the average value of efficiency in the first stage from 2008 to 2017.

The initial efficiency of environmental pollution control investments in 30 provinces and cities from 2008–2017 was measured by using the global super-efficient payoffs of scale variable SBM model, and the results are shown in columns 3, 4 of Table 2, and Figure 1.

From the inter-provincial comparison in Table 2, there are obvious inter-provincial differences in the efficiency of investment in environmental pollution control in China. Qinghai Province has the highest level of investment efficiency of environmental pollution control, ranking first with an efficiency value of 1.009; Heilongjiang Province has the lowest level of efficiency, ranking last with an efficiency value of 0.301. The investment efficiency of environmental pollution control of economically developed provinces does not rank as high as one might expect, such as Shanghai ranking eleventh and Guangdong Province ranking fifteenth. On the contrary, some of the less developed provinces ranked more highly in investment efficiency of environmental pollution control, such as Qinghai Province and Ningxia Hui Autonomous Region ranked first and second, and Yunnan and Guizhou ranked sixth and seventh, respectively. This indicates that there are various factors affecting the investment efficiency of environmental pollution treatment in each province. From an overall perspective, there are also obvious regional differences in the efficiency of governance investment. Among the four regions, the eastern region has the highest pollution control efficiency, with an average efficiency value of 0.682; the western region is the second, with an average efficiency value of 0.680; the central region has an average efficiency value of 0.637; and the northeastern region has the lowest level of control efficiency, with an average value of 0.458. By comparing the data, we can find that the efficiency values of the eastern region and the western region are close to each other and rank high; the difference between the efficiency values of the eastern region and the northeastern region is as high as 0.224. This may be due to the fact that the eastern region has developed earlier and has sufficient economic support for environmental pollution control [33,34]. The Chinese government, considering the balanced development between regions, has placed the development of the western region in a strategic position and focused on local environmental protection while carrying out the development of the western region, taking various measures to protect the ecological environment of the western region [35,36]. Therefore, the efficiency of governance has been improving year by year, and is approaching that of the eastern region. The industrial sector in the northeast has been developing since the early years, and is known as “China’s old industrial base”. These industries have caused serious environmental pollution over the years, resulting in the pollution control effect not being very obvious [37,38].

As shown in Figure 1 from the national perspective, on average, the efficiency value was always less than 1 from 2008 to 2017 without removing the influence of environmental factors and random variables, which indicates that the initial efficiency of environmental pollution control in China was always ineffective. However, with the passage of time, the overall trend shows a gradual increase. The increasing efficiency of investment in environmental pollution control is closely related to the fact that the Chinese government has been paying more and more attention to environmental pollution control over the years and actively promoting the construction and improvement of environmental protection [39,40]. The Chinese government has gradually recognized the importance of environmental protection, proposed the goal of building a beautiful China, formulated various environmental pollution control policies, and increased the scale of environmental protection investment [41,42,43]. However, the investment efficiency of environmental pollution control value has never reached 1, indicating that the effectiveness of China’s environmental pollution control is still not optimistic and there is still much room for improvement.

### 4.2. Second Stage SFA Regression Analysis

The input indicator slack variables and the three external environment variables calculated in the first stage were substituted into the stochastic frontier regression equation (Stochastic Frontier Analysis, SFA), and the results are shown in Table 3.

According to the regression results in Table 3, the slack variable coefficient of government environmental support efforts input on the four inputs is positive, which indicates that government environmental support efforts have negative impact on the four inputs. In particular, it has the greatest negative impact on the amount of investment in urban environmental pollution control infrastructure, far exceeding the other three inputs. This shows that although China has invested heavily in environmental pollution, but it is not effective and there is great deal of redundancy. Investment redundancy refers to the fact that the investment does not produce the expected results and there is a large gap between the actual input and output levels. Investment redundancy in government environmental investments is due to inefficient management. Duplication of functions between government departments, implementation of environmental policies by local agencies are still at low level, and management and planning of environmental inputs needs to be improved [44,45].

The slack variable coefficient of the local economic development level on household garbage harmless treatment plant and urban sewage treatment plants is negative, and it is significant at the level of 5%, which indicates that it has a positive impact on these two inputs. The slack variable coefficient of this variable is positive for the amount of investment in urban environmental pollution control infrastructure and the amount of investment in industrial pollution control. It indicates that the level of local economic development has negative impact on these two inputs.

The coefficients of slack variables of socialization level on the amount of investment in urban environmental pollution management infrastructure, household garbage harmless treatment plants, and urban sewage treatment plants are positive. It shows that the increase in the level of socialization is not conducive to reducing the gap between the actual and optimal levels of these three inputs. This may be due to the fact that with the increase of urbanization, many people gather to live in towns and cities, which intensifies the production of domestic waste and sewage, adding to the burden of pollution control in these areas [46]. On the contrary, this variable has positive effect on the amount of investment in industrial pollution control. This may be due to the fact that with the increase in urbanization, the population was increasingly gathering in cities, and the government has increased its efforts to control industrial pollution for the sake of people’s healthy life [47].

From the above analysis, it can be concluded that the efforts of government environmental support, the level of local economic development, and the level of socialization, have different effects on the redundancy of various inputs to environmental pollution control. In addition, in the first stage the efficiency of pollution control in economically developed provinces did not rank as high as we thought, such as Shanghai ranked eleventh and Guangdong Province ranked fifteenth. On the contrary, some less developed provinces ranked more highly in pollution control efficiency, such as Yunnan Province and Guizhou Province ranked sixth and seventh respectively. This also indicated that the calculation results of the first stage deviated from the actual situation. This may be due to the large differences in random factors in terms of area size, resources, population, industrial structure, and technological progress between different regions and provinces, which affected the accuracy of investment efficiency in environmental pollution treatment. Therefore, it is necessary to adjust the relevant data in the first stage input indicators, for example, the amount of redundancy in urban environmental pollution control infrastructure investment and industrial pollution control investment in 30 Chinese provinces and cities are adjusted, and these redundancies affect the objective evaluation of the efficiency of environmental pollution control, and, re-calculate to arrive at a more accurate value of investment efficiency in environmental pollution control.

### 4.3. Analysis of Governance Efficiency after the Third Stage of Adjustment

**Figure 2:**
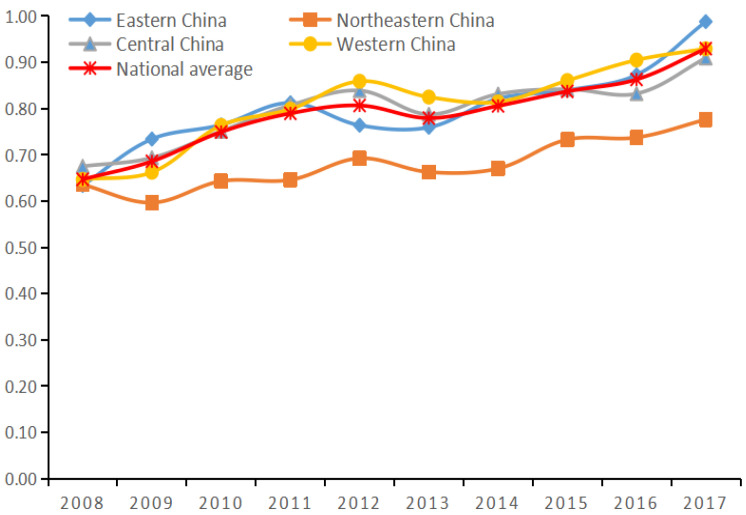
Change in the mean value of efficiency in the third stage from 2008 to 2017.

Based on the second stage Stochastic Frontier Analysis (SFA) regression equation calculations, the original input index data values were adjusted and substituted into the first stage super-efficient SBM model to produce more accurate and realistic efficiency values. The results are shown in columns 5 and 6 of Table 2 and in Figure 2. The adjusted efficiency values in the third stage show significant changes compared to the first stage.

From the overall analysis of the country (see Figure 2), after adjustment, the investment efficiency value of China’s environmental pollution control has increased significantly. The average efficiency value has increased from 0.654 in the first stage to 0.789, an increase of 20.6%. This shows that the existence of external environmental variables and random factors makes the value of investment efficiency of environmental pollution control underestimated. The average efficiency value shows a gradual upward trend in ten years, and it is gradually close to 1 compared with the first stage. This shows that China has achieved certain results in environmental pollution control in the past ten years [48]. If the growth trend continues, China’s future environmental pollution control investment efficiency will gradually reach a good state. Lu, W et al. (2022) study on the efficiency of urban green development in developing countries concluded that the efficiency of environmental pollution control investment was from 0 to 1, 0 meant no efficiency, 1 meant fully effective, if it exceeded 0.75 was a relatively high level of efficiency [49]. Therefore, the efficiency value of 0.75 or even higher is the expected level. Government plays the main role in the environmental system, because in China’s environmental governance system, government is in a dominant position [50]. The Chinese government has launched a series of treatment measures in the areas of air pollution control, water environmental protection, soil protection, and domestic pollution control. In 2016, the Chinese government conducted a hard-fought battle against environmental pollution and released the “13th Five-Year Plan for the Implementation of Environmental Impact Assessment Reform”, which focuses on improving environmental quality and enhancing the effectiveness of environmental impact assessment in a comprehensive manner [51]. In 2017, the Chinese government issued the Opinions on the Delineation and Strict Compliance of Ecological Protection Red Line, which was expected to basically establish the ecological protection red line system within three years [52]. In 2018, China introduced regulations for the Environmental Access Conditions for Domestic Waste Incineration and Power Generation Construction Projects, which can improve the efficiency of domestic waste and wastewater treatment by regulating land use for waste treatment facilities, incineration technology, project water use, waste transportation, exhaust gas pollution control measures, and equipment [53,54].

Analysis from the perspective of provinces (see columns 5 and 6 of Table 2), the gap in investment efficiency of environmental pollution control between provinces has narrowed. Among the 30 provinces studied, 24 provinces, or 80% of all provinces, have changed their investment efficiency of environmental pollution control rankings. In the first stage, Qinghai Province ranked first in investment efficiency of environmental pollution control, and after adjustment, the efficiency value of Ningxia Hui Autonomous Region improved by 3%, surpassing the first place achieved by Qinghai Province; the efficiency of Inner Mongolia Autonomous Region improved by 9 places, with an increase of 58%; Beijing dropped from the first stage ranking 25 to 30th place, with a 20% decrease in efficiency value. This shows that the development of different provinces, environmental policies and other factors affect the efficiency of environmental pollution control in each province [55]. From a practical point of view, although there is a gap in environmental investment efficiency of environmental pollution control between provinces, it is not as large as in the first phase.

From the analysis of each region (Figure 1, Figure 2 comparison), it was different from the large fluctuation in the first stage. After the adjustment, the fluctuation range of the change level of the efficiency value in each region was small. At the same time, the gap in the investment efficiency of environmental pollution control of each region has also narrowed compared with the first stage, and the regional investment efficiency of environmental pollution control has been improved to varying degrees. This shows that the influence of random factors and environmental variables reduces the actual efficiency value. The ranking of investment efficiency of environmental pollution control across regions also changed. After the adjustment, the western region’s investment efficiency of environmental pollution control ranked first, surpassing the eastern region, reaching the first place, with an average efficiency value of 0.806; the eastern region ranked second in terms of investment efficiency of environmental pollution control, with an average efficiency value of 0.799; the central region ranked third in terms of investment efficiency of environmental pollution control, with an average efficiency value of 0.797; the investment efficiency of environmental pollution control of the Ministry of Finance improved the most, with an increase of 48%, but it still ranks last, with an average efficiency value of 0.679. This may be due to the fact that although the efficiency of environmental pollution control in the eastern region was high in the past, the development speed was too fast, and the ability of environmental pollution control was far behind the speed of pollution diffusion, thus reducing the efficiency of governance in the eastern region. The western region has unique natural resources. In addition, the Chinese government attaches great importance to local environmental protection while implementing the western development policy. The introduction of large-scale funds and various supporting policies has promoted the efficiency of environmental pollution control in the western region improvement [56].

### 4.4. Analysis of GML Index of Environmental Pollution Control Investment Efficiency in China

#### 4.4.1. Overall Time Series Variation Characteristics

**Figure 3:**
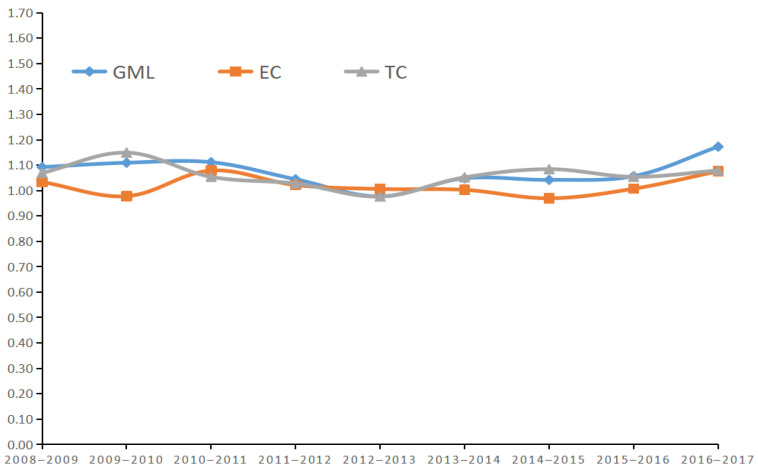
Average GML index and its decomposition line chart of each Year in China.

The DEA software (MaxDEA Software Ltd, Beijing, China) was used to find out the average GML index of investment efficiency of environmental pollution control in China for each year from 2008 to 2017 and decomposed, and the results are shown in Table 4 and Figure 3.

According to the average GML index for each year in China, it was greater than 1 for the remaining years except for 2012–2013, which was less than 1. This indicates that the efficiency of investment in environmental pollution control in China is gradually improving in general. This also coincides with the trend of efficiency values calculated in the third stage above.

As shown in Figure 3, China’s GML index shows a general “W” pattern of falling, then oscillating up, then falling and then rising again. Since 2013, the investment efficiency of environmental pollution control has started to climb year by year. This is probably due to the fact that the Chinese government has introduced several environmental pollution control policies during this period to strengthen environmental protection in various aspects. For example, the Regulations on Urban Drainage and Sewage Treatment, the Action Plan on Prevention and Control of Air Pollution, and the Opinions on Accelerating the Development of Energy Conservation and Environmental Protection Industry [57,58,59]. From the decomposition of the GML index, the overall trend of the GML index is closer to the trend of the TC index, while it is more different from the trend of the EC index. This indicates that the GML index is more obviously influenced by the TC index, while it is less influenced by the EC index. That is, the improvement of investment efficiency of environmental pollution control in China is more influenced by technological progress. Fan, M et al.’s (2018) study of industrial CO_2_ emission performance concluded that the more the number of TC (technological progress) index greater than 1, the better. All TC index (technological progress) greater than 1 was the best result and represented a significant contribution of technological progress. Currently, only one year (2012–2013) of China’s TC (technological progress) index was less than 1, and the rest were greater than 1. Therefore, in the future, the TC (technological progress) index all above 1 is the expected level to be reached [60]. The main investment in environmental pollution control is government investment. This is because enterprises are profit-seeking and do not have a strong willingness to take the initiative to invest in environmental pollution control. China has been increasing its investment in environmental pollution control, both financially and materially, with particular emphasis on the development of environmental protection science and technology [61,62]. The Chinese government has formulated and completed the 12th Five-Year Plan for the Development of Science and Technology for National Environmental Protection, and by the end of 2015, 675 achievements in basic theory, soft science and applied technology were registered with the National Environmental Protection Science and Technology Achievements [63]. China’s rapid development in environmental protection science and technology has provided a powerful boost to environmental pollution control, the technology is mainly applied to environmental pollution monitoring and prevention. Remote sensing monitoring of haze and air pollution source emissions can be carried out efficiently using satellite platforms [64], and the application of Geographic Information System (GIS) technology has strengthened the supervision of environmental pollution management [65].

#### 4.4.2. Inter-Provincial GML Index and Decomposition Index Analysis

The arithmetic mean of the annual average GML index and its decomposition results EC index and TC index for each province in China from 2008 to 2017 were used for the analysis, and the results are shown in Table 5.

Among the 30 provinces in China, the annual average GML index was greater than 1 in 29 provinces, except for Hainan Province, which was less than 1. This indicates that in general, the efficiency of investment in environmental management in Chinese provinces was improved year by year. Looking at the annual average EC index of 30 provinces, 18 provinces were greater than 1, accounting for 60%. In terms of the annual average TC index of 30 provinces, 28 provinces were greater than 1, accounting for 93%. This indicates that the improvement of investment efficiency in environmental pollution control in Chinese provinces is mainly due to the progress of self technology.

#### 4.4.3. Regional GML Index and Decomposition Index Analysis

**Figure 4:**
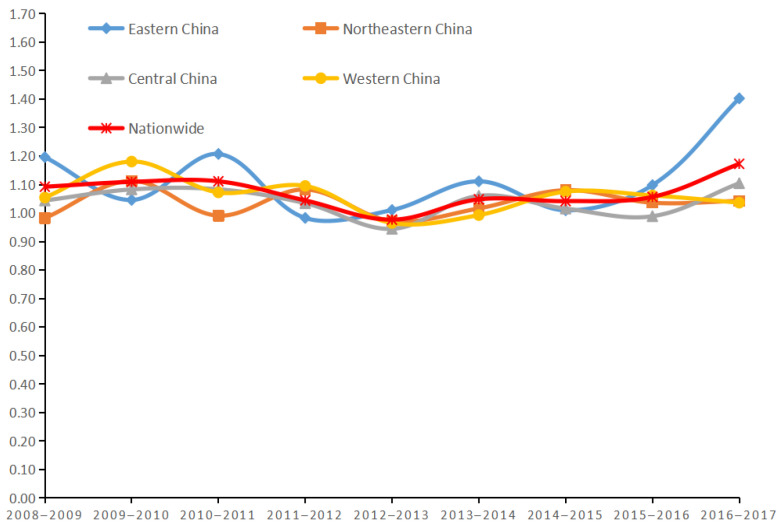
Trends in the annual average GML index in the four regions from 2008 to 2017.

Based on the data analyzed above, the trend of the annual average GML index for the four regions of China from 2008 to 2017 was derived based on the GML index, as shown in Figure 4.

The change trend of the annual average GML index in the four regions from 2008 to 2017 was similar, and all of them were in oscillating and fluctuating state. The trend of change in the central region was basically consistent with the national trend, with a slow rise during 2008–2011, and began to decline after 2011, reaching the lowest in 2013 with an average value of 0.945, and then a fluctuating upward trend, reaching the highest in 2017 with an average value of 1.105, and generally speaking, the efficiency of investment in environmental pollution control has improved. The western and northeastern regions fluctuate in a basically consistent state, and the overall was in an upward trend. The eastern region has the largest fluctuation. During the period of 2008–2015, it showed repeated fluctuations of falling, then rising, and then falling, and after 2015, it gradually showed an upward trend and reached the highest in 2017, with a mean value of 1.402, and the overall efficiency has improved. In order to clarify the reasons for the changes in the efficiency of environmental pollution control investment in each region, the annual average GML index of the four regions is also decomposed, and the results are shown in Table 6.

Overall, the EC index and TC index of the four regions were both higher than 1 during the decade, which indicates that both the efficiency and technology of environmental China pollution control investment have improved during this period. The TC indexes of all four regions were greater than the EC indexes, which indicates that the improvement in the efficiency of environmental pollution control investment in each region was mainly due to the progress in technology. In terms of the ten-year national average, the EC index was greater than 1 in 2009, 2011, 2012, 2013, 2014, 2016, and 2017, indicating that the improvement in technical efficiency during the period was conducive to the improvement in the efficiency of environmental pollution control investment. In contrast, the TC index was higher than 1 in all years except 2013 when it was less than 1, indicating that the improvement of technology contributes significantly to the improvement of investment efficiency in environmental pollution control in the rest of the period except 2013. The TC index was higher than 1 for the three regions of East, Central, and West except for three years in 2011, 2012, and 2013, which indicates that for these three regions, the improvement in the efficiency of investment in environmental pollution control was more obvious due to the improvement of technology.

## 5. Discussion

The conclusions drawn from the panel data of China’s environmental pollution control investment from 2008–2017 can provide a reference for China’s control of environmental pollution, but there are still some shortcomings that need to be further improved. First, such as the data collection is not very comprehensive. The panel data of Hong Kong, Macau, Taiwan, and Tibet Autonomous Region provinces can be complemented by software model simulations in the future, and also the research years can be extended. Second, this study analyzes the investment efficiency of environmental pollution control in China and each region from the macro level, without considering the special situation of different regions, and more in-depth analysis and research can be conducted in the future for the characteristics of different regions and explore the related influencing factors. For example, the eastern region has a developed economy and a large population, which generates more environmental pollution, but why is the environmental pollution control efficiency high [66,67]? Assuming that the possible reasons are sufficient capital, advanced technology and concentration of talents, what is the magnitude of the contribution of these factors to environmental protection? What is the root cause of the inefficiency of environmental pollution control in the northeast region compared to other regions, and how can this problem be addressed [68,69]?

## 6. Conclusions

This paper analyzes the investment efficiency of environmental pollution control in China from 2008 to 2019 by using the three-level super-efficiency SBM-DEA model and the GML index, and draws the following conclusions: First, the investment efficiency of environmental pollution control in China is improving, but there are obvious disparities among different regions. Affected by random factors and environmental variables, the actual efficiency value decreases. Second, excluding the effects of both, the national investment efficiency of environmental pollution control has improved significantly, but still has not reached the optimal effect. For example, after adjustment, the average efficiency of environmental pollution control in China has increased from 0.654 in the first stage to 0.789 in the third stage, with an increase of 20.6%, but the efficiency is always less than 1. At the same time, the gap between provinces and regions has narrowed while the investment efficiency of environmental pollution control has improved, and there is still an unbalanced situation. For instance, the average efficiency of the western region was far higher than that of the northeast region, which is 0.806 and 0.679 respectively. Third, overall, China’s environmental pollution control efficiency has improved year by year, and the main driver is technological progress; compared with northeastern China, technological progress has a more significant role in promoting eastern, central, and western China.

Based on the above research conclusions, in order to promote the construction of environmental pollution control in China, the following suggestions are put forward:

(1) Strengthen regional cooperation to jointly control environmental pollution [70]. From the panel data of 30 Chinese provinces, it can be concluded that there is a regional development imbalance in the efficiency of investment in environmental pollution control in China. In order to achieve the improvement of overall environmental pollution treatment efficiency, the eastern region can export advanced environmental protection technology to the central and western regions, and the western region can use its abundant natural resources to cooperate with the eastern region. Other regions should lend a helping hand to the northeast region by sharing environmental governance experience, advanced environmental technologies, etc., and exporting environmental governance talents [71].
(2) Vigorously develop the rural economy. As concluded in the previous article, with the increasing level of urbanization, the concentration of residents living in towns and cities has intensified the generation of domestic waste and sewage. The development of rural economy can relieve the pressure of environmental pollution management caused by the concentration of urban population. In the process, attention should also be paid to the environmental protection of rural areas [72].
(3) Environmental protection investment should be targeted [73]. From the results of the impact analysis of the external environment, the Chinese government’s annual funding for environmental pollution control is increasing, but the positive impact on various output indicators has not improved. Therefore, environmental protection investment should be targeted to prevent investment redundancy.
(4) Increase support for environmental protection technology research. According to the GML index and its decomposition, the main reason for the annual increase in the investment efficiency of China’s environmental pollution control is technological progress. Therefore, it is necessary to increase support for the research and development of environmental protection technology, such as introducing policies that are conducive to the development of the environmental protection technology industry, increasing the research and development funds of environmental protection technology, and cultivating talents in the field of environmental protection [74,75,76,77].

## Figures and Tables

**Table 1:** Investment efficiency indicators of environmental governance.

Tier 1 Indicators	Tier 2 Indicators	Tier 3 Indicators
Input variables	Financial input	Industrial pollution control investment (billion yuan)
		Urban environmental pollution management infrastructure investment amount (billion yuan)
	Material input	Household garbage harmless treatment plants (seat)
		Urban sewage treatment plants (seat)
Output variables	Industrial emissions treatment	general solid waste comprehensive utilization (million tons)
		The number of industrial waste gas pollution treatment facilities (sets)
	Living pollution treatment	Household garbage harmless treatment rate (%)
		Urban sewage treatment rate (%)
Environmental variables	Government environmental support efforts	The proportion of environmental pollution treatment investment in GDP (%)
	Local economic development level	GDP (billion yuan)
	Socialization level	Urbanization rate (%)

**Table 2:** Efficiency averages and rankings by province from 2008 to 2017.

Region	Province	Stage 1		Stage 3	
		Average Efficiency	Ranking	Average Efficiency	Ranking
Eastern China	Beijing	0.443	25	0.534	30
	Tianjin	0.852	8	0.904	7
	Hebei	0.977	3	0.986	5
	Shanghai	0.727	11	0.792	15
	Jiangsu	0.490	22	0.719	21
	Zhejiang	0.665	13	0.760	18
	Fujian	0.549	19	0.739	19
	Shandong	0.640	16	0.817	14
	Guangdong	0.655	15	0.736	20
	Hainan	0.956	5	1.004	2
Northeastern China	Liaoning	0.684	12	0.894	8
	Jilin	0.388	28	0.582	28
	Heilongjiang	0.301	30	0.562	29
Central China	Shanxi	0.956	4	0.991	4
	Anhui	0.768	10	0.885	9
	Jiangxi	0.658	14	0.803	14
	Henan	0.575	18	0.767	16
	Hubei	0.389	27	0.658	25
	Hunan	0.474	23	0.677	24
Western China	Nei Monggol	0.533	20	0.842	11
	Guangxi	0.581	17	0.769	17
	Chongqing	0.833	9	0.869	10
	Sichuan	0.495	21	0.694	22
	Guizhou	0.866	7	0.822	12
	Yunnan	0.946	6	0.941	6
	Shaanxi	0.436	26	0.692	23
	Gansu	0.449	24	0.600	27
	Qinghai	1.009	1	0.998	3
	Ningxia	0.993	2	1.018	1
	Xinjiang	0.341	28	0.626	26

**Table 3:** Results of the second stage stochastic frontier regression.

	Redundant Investment in Industrial Pollution Control		Redundant Investment in Urban Environmental Pollution Management Infrastructure	
	Coefficient	Standard Deviation	Coefficient	Standard Deviation
Constants	−0.406374 *	−3.848926	−194.687790 ***	47.858103
government environmental support efforts	2.302858 ***	0.925156	70.570078 ***	8.950301
local economic development level	0.000153	0.000058	0.001269 **	0.000511
socialization level	−0.091174 *	0.066246	1.091758 *	0.716531
sigma-squared	106.984520 ***	20.322746	10011.500000 ***	1.437268
gamma	0.295561 **	0.127572	0.411285 ***	0.052723
loglikelihoodfunction	−1094.0241		−1751.6127	
LR one-sided error	46.32686 ***		30.99629 ***	
	**Household Garbage Harmless** **Treatment Plant Redundancy**		**Urban Sewage Treatment** **Plant Redundancy**	
	**Coefficient**	**Standard Deviation**	**Coefficient**	**Standard Deviation**
Constants	−3.5224076 *	3.8623349	−23.451946 **	13.765248
government environmental support efforts	0.22772769 *	0.60479266	2.6279131 *	1.9546761
local economic development level	−0.00017399 **	0.000070359	−0.000456998 **	0.000256776
socialization level	0.059836647 *	0.064210501	0.36500775 *	0.2223983
sigma-squared	200.45879 ***	72.527272	1578.6747 **	632.2929
gamma	0.87481371 ***	0.048841983	0.83143444 ***	0.072676693
loglikelihoodfunction	−955.29407		−1308.6314	
LR one-sided error	144.03577 ***		168.24658 ***	

Note: “***”, “**” and “*” indicate significant at the 1%, 5% and 10% levels, respectively.

**Table 4:** Average GML index and its decomposition by year in China.

Year	GML Mean Value	EC Mean Value	TC Mean Value
2008–2009	1.110	1.034	1.069
2009–2010	1.111	0.978	1.149
2010–2011	1.111	1.079	1.054
2011–2012	1.044	1.022	1.028
2012–2013	0.977	1.006	0.977
2013–2014	1.048	1.003	1.052
2014–2015	1.042	0.970	1.084
2015–2016	1.057	1.007	1.054
2016–2017	1.173	1.076	1.079

**Table 5:** Average annual GML index and its decomposition by province from 2008 to 2017.

Region	Province	GML	EC	TC
Eastern China	Beijing	1.475	1.170	1.232
	Tianjin	1.130	0.999	1.130
	Hebei	1.037	1.001	1.037
	Shanghai	1.143	1.001	1.131
	Jiangsu	1.057	0.995	1.063
	Zhejiang	1.123	0.992	1.112
	Fujian	1.059	0.982	1.070
	Shandong	1.060	0.999	1.062
	Guangdong	1.098	1.080	1.105
	Hainan	0.999	0.995	1.004
Northeastern China	Liaoning	1.010	0.999	1.012
	Jilin	1.046	1.017	1.064
	Heilongjiang	1.049	0.995	1.111
Central China	Shanxi	1.002	1.009	0.994
	Anhui	1.041	0.996	1.045
	Jiangxi	1.075	1.006	1.071
	Henan	1.022	0.998	1.041
	Hubei	1.045	1.062	1.025
	Hunan	1.054	1.028	1.032
Western China	Nei Monggol	1.046	0.998	1.048
	Guangxi	1.058	0.999	1.060
	Chongqing	1.064	0.998	1.065
	Sichuan	1.021	1.010	1.020
	Guizhou	1.096	1.028	1.070
	Yunnan	1.013	1.005	1.010
	Shaanxi	1.051	1.002	1.060
	Gansu	1.177	1.083	1.103
	Qinghai	1.003	1.035	0.974
	Ningxia	1.002	0.999	1.004
	Xinjiang	1.122	1.095	1.064

**Table 6:** Decomposition results of the average GML index by year for the four regions.

Index	Region	08–09	09–10	10–11	11–12	12–13	13–14	14–15	15–16	16–17	Mean
EC	Eastern China	1.003	0.942	1.105	0.995	1.003	0.991	0.933	0.995	1.226	1.021
	Northeastern China	1.015	1.048	0.866	1.253	0.898	1.132	0.863	1.088	0.869	1.004
	Central China	1.011	0.960	1.131	0.968	1.019	1.011	1.025	0.957	1.067	1.017
	Western China	1.079	1.001	1.084	1.011	1.032	0.973	1.002	1.023	1.000	1.023
	Whole country	1.033	0.978	1.079	1.022	1.006	1.003	0.970	1.007	1.076	1.019
TC	Eastern China	1.193	1.138	1.131	0.981	1.007	1.122	1.087	1.098	1.097	1.095
	Northeastern China	0.965	1.074	1.179	0.878	1.103	0.928	1.254	0.963	1.217	1.062
	Central China	1.036	1.131	0.970	1.077	0.927	1.053	1.009	1.054	1.055	1.035
	Western China	1.003	1.190	0.995	1.084	0.941	1.022	1.076	1.039	1.039	1.043
	Whole country	1.069	1.149	1.054	1.028	0.976	1.052	1.084	1.054	1.079	1.061

## Data Availability

Data available in a publicly accessible repository that does not issue DOIs. Publicly available datasets were analyzed in this study. This data can be found here: [https://navi.cnki.net/knavi/yearbooks/YINFN/detail, accessed on 4 April 2022] [https://navi.cnki.net/knavi/yearbooks/YHJSD/detail, accessed on 4 April 2022].
